# Defining Food Safety Inspection

**DOI:** 10.3390/ijerph19020789

**Published:** 2022-01-11

**Authors:** Jason Barnes, Harriet Whiley, Kirstin Ross, James Smith

**Affiliations:** College of Science and Engineering, Flinders University, Bedford Park, SA 5042, Australia; harriet.whiley@flinders.edu.au (H.W.); kirstin.ross@flinders.edu.au (K.R.); jim@jamescsmith.com (J.S.)

**Keywords:** food safety, inspection, health protection, meaning attribution, constructionism, consumer, food business associate, inspector, compliance

## Abstract

Food safety inspections are a key health protection measure applied by governments to prevent foodborne illness, yet they remain the subject of sustained criticism. These criticisms include inconsistency and inadequacy of methods applied to inspection, and ineffectiveness in preventing foodborne illness. Investigating the validity of these criticisms represent important areas for further research. However, a defined construct around the meanings society attributes to food safety inspection must first be established. Through critical examination of available literature, this review identified meanings attributed to food safety inspection and explicates some of the key elements that compose food safety inspection as a social construct. A total of 18 meanings were found to be attributed to food safety inspection. Variation in meanings were found between consumers, food business associates and food safety inspectors. For some, inspection meant a source of assurance, for others a threat to fairness, while most view inspection as a product of resources and inspector training. The meanings were then examined in light of common criticisms directed at food safety inspection, to expound their influence in how food safety inspection is realized, shaped, and rationalized. This review highlights the influence of sociological factors in defining food safety inspection.

## 1. Introduction

Foodborne illness presents a significant public health challenge worldwide [1]. Quantifying the impact of foodborne illness is difficult due to the varying effectiveness of public health surveillance systems between countries, the influence of co-morbidities, the uncertainty that comes with under-reporting and diagnosis, access to health care, and the individual experience of illness [1,2]. The World Health Organization [3] estimates 600 million cases of foodborne illness were experienced worldwide in 2010, leading to an estimated 420,000 deaths. While these figures represent imprecise estimates, they offer some suggestions to how foodborne illness remains one of the leading causes of death worldwide, particularly in developing nations.

To combat foodborne illness, many governments have introduced health protection measures and food safety regulatory systems. Food safety inspection is a health protection method employed by many food safety regulators worldwide [4]. Food safety inspection can take various forms when applied at different stages of the food production system. In some instances, the term inspection is synonymous with analysis or examination [5]. Here, food safety inspection involves the direct analysis of a sample of food to establish details on its composition, level of contamination or quality. This form of food safety inspection is often applied where foods are being imported, or prior to foods entering a consumer market [5]. More traditionally, food safety inspection involves assessment of food handling practices and the condition of food production environments [6]. This style of inspection is common to the food retail sector, but it can also be applied to food manufacturing and primary production environments such as farms and slaughterhouses [7]. To undertake this style of inspection, often inspectors will attend food production premises and apply observational and qualitative approaches to establish details on food handling practices in place and determine whether these practices put food at risk of contamination [8].

The approach to undertaking food safety inspection also varies considerably between jurisdictions. Some jurisdictions implement a framework of prescriptive food safety requirements for food businesses, applying inspection as a means of measuring a food businesses’ extent of compliance with those criteria [8]. This compliance-check approach is often accompanied by an inspection checklist and a simplistic numerical scoring or grade system [7,9]. These scoring systems can be weighted where more points are deducted for some non-compliances over others, or can be designed to determine maximum scores that can be achieved where some specific non-compliances are observed [10]. Where a scoring system is applied, regulatory systems often prescribe actions to be taken by the regulator in response to score ranges, such as enforcement, or ongoing inspection frequency [4,8,11]. Compliance-check systems may also be in place where the regulatory system includes public disclosure of inspection results [10]. The compliance-check approach to food safety inspection requires little autonomy and determination by inspectors as it generally provides binary options for compliance assessment and prescribes the actions to be taken by regulators following their observations [6].

In contrast, food safety inspection can also be applied as a qualitative risk assessment. This inspection approach is usually accompanied by food safety requirements that are broad, outcome-focused food safety objectives [12]. Inspectors are often guided by established risk assessment frameworks such as Hazard Analysis and Critical Control Point planning (HACCP), or other proprietary risk assessment frameworks in their examination of food handling practices and conditions [12]. These systems require inspectors to apply autonomy and professional judgement in the assessment process and place much of the responsibility for determination of inspection results and subsequent regulatory actions with the inspector.

Although the two approaches to food safety inspection described here are distinct extremes in their method and philosophy, inspection approaches applied by regulators tend to fall into one of the countless positions on the continuum between them. Further variation can be observed between jurisdictions and their regulatory systems in the way food safety inspection is applied to prevent foodborne illness. For some jurisdictions such as Counties and Local Governments in California [13], Ohio [14], Singapore [15], and across the United Kingdom [16], food safety inspection is a means to establishing transparency and driving market pressures by creating incentives for compliance via disclosure of inspection scores or reports to the public [6,17]. However, disclosure of inspection results has been shown insufficient to improve food safety standards when it is applied in isolation [18]. Traditionally, food safety inspection has been applied to identify and report food safety deficiencies to food business operators to resolve, often within a specified timeframe. As inspection is usually undertaken within a regulatory framework, compliance motivators such as monetary penalties and other enforcement measures may also be determined and initiated by inspection findings [6]. The primary distinction in how food safety inspection is applied by regulators appears to be whether it is applied as a preventive measure or a reactive measure.

While food safety inspection is a key health protection measure applied by governments to prevent foodborne illness, it is rarely applied in isolation of other measures and remains the subject of sustained criticism. Key criticisms include inconsistency, ineffectiveness in identifying and preventing foodborne illness, and inadequacy of methods applied to inspection [6,12,19]. These criticisms represent important areas for further research, yet in order for this to be holistic and significant, further depth must first be established around how society views food safety inspection, and the meanings society attributes to food safety inspection. Accordingly, these common criticisms may themselves be viewed as social constructs, and be the subject of social interactions that lead to their identification as problems [20].

Without a clear understanding of why food safety inspection has been adopted as a key method of health protection, and what society expects food safety inspection to achieve, attempts to improve methods of inspection or measure the effectiveness of food safety inspection will be less successful. Adopting such a perspective attempts to establish truths on the basis of assumptions and ignores complex and critical social mechanisms that define, shape and characterize food safety inspection. In absence of the understanding of the meanings attributed to food safety inspection and its significance and interplay in society, criteria for comparison and evaluation will remain incomplete. Hence, establishing the meanings attributed to food safety inspection is an imperative pre-requisite to any further significant research into food safety inspection and its effectiveness or method.

This review examined what food safety inspection means to society, what society expects to gain from inspection, and determines whether the understanding and experience of food safety inspection differs amongst those engaged in the process. It identified the influences that shape these interpretations, particularly with regard to what these groups believe inspection achieves, and how these groups shape these explanations of causality. Hence, this review adopts a position reflective of social constructionism, whereby meaning is deemed to be knowledge constructed by society through interpretation and lived realities [21]. This applies an epistemological stance that knowledge is held in the beliefs and interpretations of members of society and that these are realities, regardless of their objectivity [22].

Through critical examination of available literature, this review sought to identify meanings attributed to food safety inspection, and explicate some of the key elements that compose food safety inspection as a social construct. The values, constructs and meanings attributed to food safety inspection are then examined in light of common criticisms directed at food safety inspection, to expound their influence in how food safety inspection is realized, shaped and rationalized.

## 2. Materials and Methods

A search protocol was crafted using an adaptation of the SPIDER [23] search tool. Some search terms were adopted from Cooke, Smith and Booth [23] while others were identified through prior exploration of the literature. The search protocol was rationalized to include only search terms relating to the phenomenon of interest and evaluation with the intention to avoid any unnecessary limitation. The search terms used were “((“Food-safety-inspection*” OR “Restaurant-Inspection*” OR “Hygiene-Inspection*” OR “Food-safety-assessment*” OR “Food-safety-audit*”)) AND ((meaning OR view OR experience OR opinion OR attitude OR perception OR belief OR feeling OR knowledge OR understanding))”. Some minor modifications were made to the search terms where online databases imposed limits on search terms.

Academic journal articles and grey literature were sought from six online databases: ProQuest, Scopus, Informit, Medline (OVID), Science Direct (Elsevier), and Web of Science, using the predefined search protocol to inform this review.

Inclusion criteria were formulated in extension of the search protocol and were applied in two stages. Firstly, to the title and abstract screening phase where they guided inclusion based on participant characteristics, focus characteristics, methodological characteristics, and outcome and measure characteristics. Later, the criteria were applied to the full text screening phase in a more judicious manner, particularly in examining articles on whether they presented views or perceptions toward food safety inspection, whether those views or perceptions were drawn directly from participants or the author, and whether those views or perceptions are toward food safety inspection or attributes immediate to the construct. While the majority of articles were excluded as they did not offer beliefs or perceptions toward food safety inspection, some were excluded because the voices of respondents had been lost where results had been homogenized, while others were excluded due to the constriction of expression imposed on respondents by their survey design.

## 3. Results

**Figure 1:**
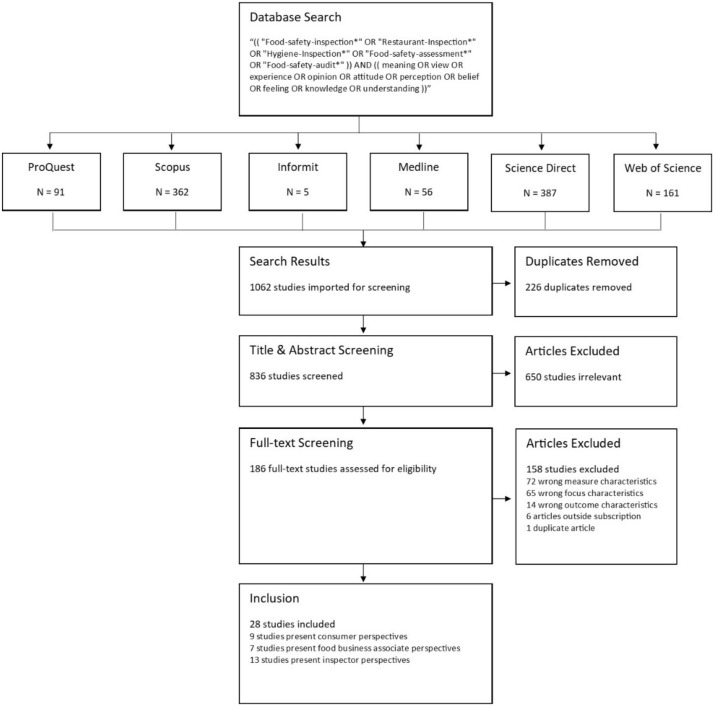
Review flow diagram.

Of the 1062 results yielded from the search, 226 were removed as they were duplicates, a further 650 articles were removed following title and abstract screening, and 158 articles removed via full text screening using a documented four-point inclusion criteria. A total of 28 articles deemed to meet the inclusion criteria have been used to inform this review (Figure 1).

**Figure 2:**
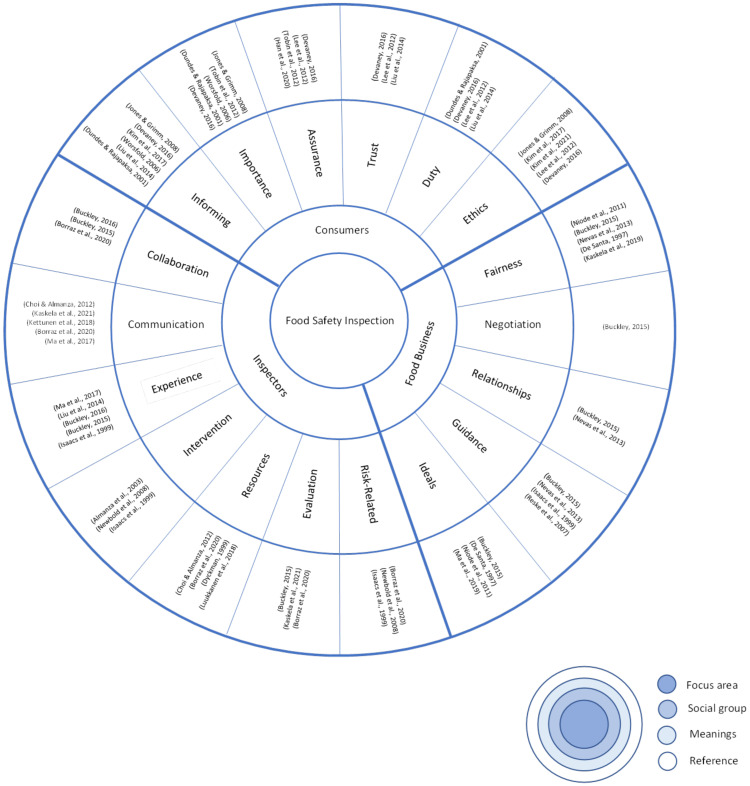
Meanings and sources.

This review identified six meanings that consumers attribute to food safety inspection, five attributed by food business associates, and seven attributed by inspectors. These meanings were drawn from the 28 articles used to inform the review. Figure 2 shows the meanings that consumers, food business associates and food safety inspectors attribute to food safety inspection, and the sources that each meaning was derived from. Some articles contributed to understanding of only one meaning while other articles offered greater versatility and breadth in the number of meanings they inform.

**Figure 3:**
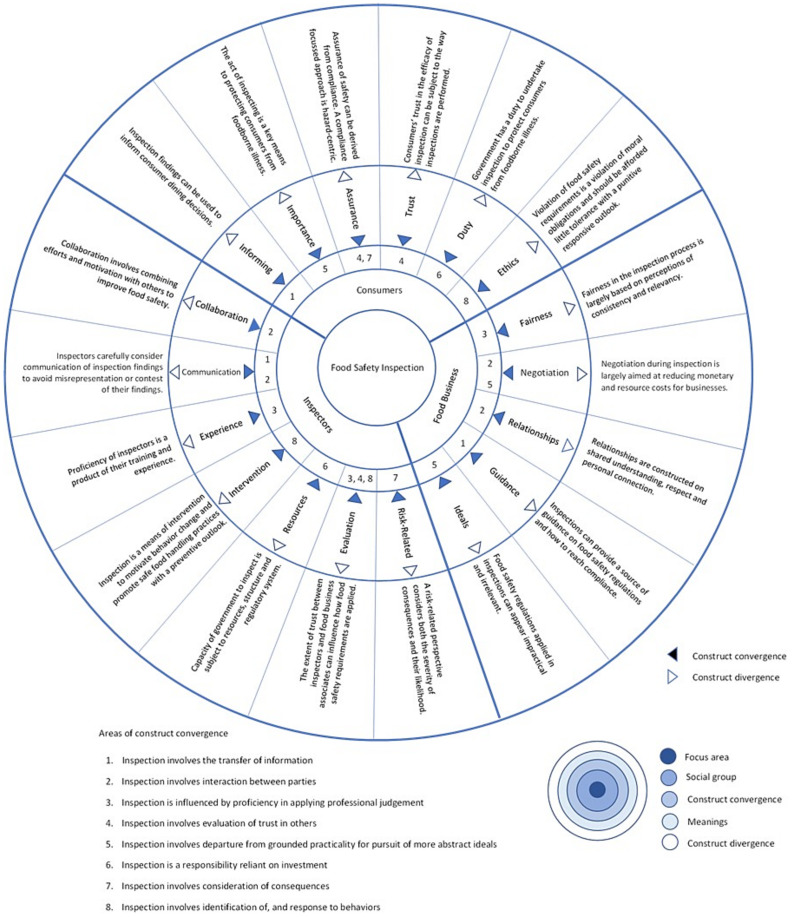
Meanings and conceptual relationships.

The meaning of inspection varies considerably between consumers, food business associates, and food safety inspectors and given this division of views, the results are presented grouped into those attributable to consumers, food business associates, and food safety inspectors. To aid in the categorization and derivation of meanings identified in this review, each meaning was examined for points of commonality and difference with others. Figure 3 provides an overview of this analysis, depicting points of conceptual convergence and divergence. In the analysis, each meaning was found to share a point of commonality with at least one other meaning. This sharing of commonalities has been referred to here as construct convergence. The categorization and ultimate determination of category boundaries was established where points of variation between meanings were found sufficient for them to be experienced or interpreted differently. These points of variation are referred to here as construct divergence.

### 3.1. Consumers

#### 3.1.1. Inspection as a Matter of Importance

For consumers, food safety inspection is viewed as an important intervention for their protection from consuming unsafe food [24,25]. While food safety inspection methods and the extent of intervention varies considerably between jurisdictions, consumers have reported a preference for inspections to be performed on a highly regular basis. In her research, Worsfold [26] found the majority of consumers expected food safety inspection to occur more than once per year, while Jones and Grimm [24] identified that 53% of consumers felt that food safety inspections should be performed at food establishments 12 or more times per year. Dundes and Rajapaksa [27] found a similar consumer preference for food safety inspection to be undertaken several times per year and be accompanied by other food safety investigation methods such as microbiological analysis of food samples. Yet while consumers nominate food safety inspection as a matter of importance and rationalize an intensive schedule by which this perceived important function should occur, the basis on which consumers establish this sense of importance is less clear. Furthermore, the manner by which consumers conceptualize a relationship between food safety inspection and their protection from consuming unsafe food was found by the authors of this review to be largely unchartered. Instead, the perception of importance of food safety inspection for consumers may be due to how inspections respond to other needs and values beyond protection such as assurance, duty, and trust [28]. This is discussed in detail below.

#### 3.1.2. Inspection as an Assurance

Increasingly, in many jurisdictions food safety inspection systems have been designed to disclose inspection results to consumers [28]. For some consumers, the disclosure system is an important source of assurance that the establishment’s compliance with food safety regulations meets their expectations and risk appetite [29]. However, reliance on disclosure systems for assurance is not universal for all consumers. Assurance for consumers can be drawn simply from the knowledge that food safety inspection is occurring [25]. Han et al. [30] identified that in Chinese provinces where more food safety inspections in the form of sample analysis had occurred, consumer perceptions of food safety were also higher. Although the knowledge of inspection scores is necessary for some consumers to derive assurance, for others assurance can be derived from knowing an inspection system is in place [25,28]. Comparison of these different sources of assurance also highlights a distinction in roles assumed by stakeholders, particularly with regards to decision making and risk assessment.

#### 3.1.3. Inspection as an Informant for Decisions

Food safety inspection can provide a source of information to consumers and other stakeholders alike. How this information is presented is subject to the design of the food safety regulatory system. For some jurisdictions, disclosure of inspection results is performed by requiring signage at the food premises, while other jurisdictions publish inspection results in print and digital media [24]. Although most consumers report not using inspection results to inform their decision on where to dine, consumers present an overwhelming desire to freely access food safety inspection reports [26,28,31]. In contrast, Jones and Grimm [24] found that 62% of respondents in their study had made dining decisions based on disclosed inspection results. For those consumers that regularly rely on disclosed food safety inspection results, it is clear the responsibility of decision making remains with them. However, for the consumers that do not rely on disclosed inspection results, the basis or responsibility of decision-making lies elsewhere. For some consumers, decision making is informed by other factors ahead of food safety results, such as concern about pesticide residuals, adulterants and food additives [32]. For other consumers, the role of decision making is viewed as a responsibility of the regulator. According to Dundes and Rajapaksa [27], 71% of consumers preferred that the regulator determine whether businesses provide safe food by allowing them to remain open, or closing premises that are unsafe, in contrast with 29% of consumers opting to make their own decisions based on disclosed inspection results.

#### 3.1.4. Inspection as a Duty of Government

Food safety inspection is widely viewed by consumers not just as a role, but as a duty of governments. The administration of food safety inspection preferred by consumers have been found to be regular in-depth investigations throughout the year coupled with microbiological analysis of food samples [27]. Some consumers prefer even stronger intervention measures including prohibition of some high risk foods and consideration of food safety threats beyond microbiological contamination [27]. Consumers expect that inspections will be performed without prior notice to the food business to ensure accuracy; they also seek transparency and regularity of the process [28,29]. There remains little question that many consumers are dissatisfied with the approaches, transparency and frequency of food safety inspections performed by governments, with their reports of skepticism on the effectiveness and accuracy of these systems [28,29,32]. This situation highlights the disparity between consumer expectations of a functional food safety inspection system and the government resources available to deliver it.

#### 3.1.5. Meaning of Inspection within a Deontological Ethical Conviction: Inspection as an Administration of Justice

Consumer expectations also contribute to a broader perspective that governments have a duty to protect consumers, that consumers have rights to protection from unsafe food, and where food producers are seen to deviate from food safety standards, they not only violate statute but violate a moral obligation to the consumer. Consumer tolerance for food business non-compliance is considerably lower than that of inspectors and food business operators [24,29,31,33]. Many consumers feel that governments are not strict enough with enforcement following food safety inspections findings [29], and that the food industry show a fearlessness toward the powers of regulators [28]. This view suggests that consumers apply an ethical lens to food safety inspection that requires strict adherence and compliance with rules and moral obligations, rather than evaluating right from wrong based on the outcomes or intention of the actions as other stakeholders do. This epistemic incongruence may be a force in determining the level of trust afforded to inspectors, food businesses, and the food safety regulatory system by consumers.

#### 3.1.6. Inspection Attributes as Antecedents to Trust: A Process Reticent in Inaccuracy, Corruption and Manufactured Transparency to Some

Trust is positioned as a determinant of significance for all meanings attributed to food safety inspection by consumers. Without trust, the utility of food safety inspection to consumers is diminished. For consumers, the apportionment of trust toward food safety inspection appears to vary, with some consumers expressing dismay and discontent toward the methods applied for food safety inspection. Trustworthiness and transparency is diminished for some consumers on the basis of announced inspections, giving opportunity for food businesses to make adjustment and correction prior to inspection [28]. Other consumers have expressed concern to the frequency of inspections, the aptitude of inspectors, the translation of inspection findings into enforcement, and efficiency of food safety inspection [28,29,32]. This may offer some explanation for the desire of consumers to have access to food safety inspection reports, even though the reliance on food safety inspection results for decision making varies between consumers. Furthermore, trust may be reflected by consumer preferences in responsibilities for decision making following food safety inspection. Consumers with a diminished trust in food safety inspection may be those taking control of decision making in dining choice, while others demonstrate a willingness to place trust in regulators to make decisions on their behalf. Overall, consumers express a clear desire for transparency, strictness, regularity and access to information to strengthen their trust in food safety inspection [28].

### 3.2. Food Business Associates

The perspectives attributed to food safety inspection by food business operators, employees, and advocates, herein referred to as food business associates, differs markedly from those of consumers.

#### 3.2.1. Inspection as a Source of Guidance

For food business associates, food safety inspection presents a personal interaction with food safety inspectors. The interaction can be transactional in nature, exchanging knowledge and concession between food business associates and inspectors [34]. Inspection can form a basis for meaningful discourse between food business associates and food safety inspectors in methods for compliance and food safety requirements [34]. Food business associates mostly view actions they take following food safety inspections as enhancing the safety of their food, and the overall hygiene of their facilities [35]. For food business associates, inspection can act as a source of food safety guidance, education and advocacy [34]. Isaacs et al. [36] found that food business associates saw inspection as their first source for food safety advice. Where inspections are announced, food business associates widely view these inspections as more opportune to establish a relationship with the inspector, to make improvements to food handling practices, and derive better understanding of the importance of food safety standards [37]. Yet while food business associates see food safety inspections as a valuable source of guidance, they also stand to experience negative consequences from food safety inspection. This is presented below.

#### 3.2.2. Inspection as Threat to Fairness, Equality, Consistency

As with all regulatory activity, consequences stand for those found failing to comply. Some food business associates report the difficulty of maintaining compliance where they observe food safety requirements regularly changing [38]. Furthermore, differences in opinion between inspectors can also mean inconsistent expectations and inconsistent focus on particular food safety issues [38]. This can also lead to frustration and confusion for food business associates where varying inspector interpretations have led to some food business being permitted to undertake an activity, while other food businesses are not [34]. In contrast, Nevas, Kalenius, and Lundén [35] found 80.1% of food business associates responding to their study felt that food businesses were met with equal demands of regulators in the immediate geographical area. While failure to comply with statutory requirements carries consequences, the consequences borne by food businesses from food safety inspection are deeper reaching. These can include regulatory burden, financial costs imposed by governments, and enduring what appear to be illogical regulatory interventions devised to manufacture transparency for the benefit of governments [39]. Food business associates also question fairness in the representation of food safety inspection results, particularly where the assessment framework does not align with their own evaluation [40]. In these circumstances, however, some food business associates report that maintaining a personalized relationship with inspectors can offer an opportunity to challenge and negotiate over matters where they do not agree [34].

#### 3.2.3. Inspection as a Foundation to Relationships

Some food business associates see food safety inspections as an opportunity to establish a personable relationship with the inspector [34]. By establishing a relationship with inspectors, food business associates report that inspectors develop greater familiarity with food production at the premises, can offer more prioritized assessment of non-compliance, offer knowledge, advice, and expertise, adopt roles as an advocate for food business associates with other parties, share an understanding of collaboration, afford food business associates more tolerance and flexibility in approaches to food production, allow food business associates to explore cheaper alternatives to compliance, and accept the input of food business associates when determining non-compliance and timeframes for rectification [34]. Nevas, Kalenius and Lundén [35] also found that where inspectors have a greater familiarity with food production processes at a premises, food business associates report an increased sense of fairness and understanding of the non-compliances to be rectified. While food business associates stand to benefit from establishing relationships, power remains with the inspector. In recognizing this, food business associates report an intention not to antagonize inspectors and accommodate them by allowing inspectors to feel like an expert and by making concessions such as not challenging some judgements even though they disagree [34].

#### 3.2.4. Inspection as a Negotiation

Inspection can take the form of a negotiation where a fitting relationship has been established with the inspector. To facilitate this exchange, some food business associates report making concession and undertaking works prescribed by the inspector on some occasions in order to negotiate more substantial items in the future [34]. For others, these negotiations take the form of alternative or non-conventional means to food business set-up and production methods. Here, negotiation on alternative methods can lead to considerable monetary savings and overcome financial barriers to commencing operation [34]. Negotiation may also occur between inspectors and food business associates where food safety regulations introduce unnecessary complexity or illogical requirements for food business operations.

#### 3.2.5. Inspection as an Application of Abstract Ideals

A particular challenge presented by inspection for food business associates is the apparent impracticality and irrelevance of food safety regulations. Food safety inspection assesses their premises and practices against food safety requirements that food business associates can view as abstract and reflecting ideals rather than realistic objectives. For some food business associates, this deviation from dealing in experience and practicality is acknowledged and endured, but for others it can lead to considerable expense, impracticality, and complication of food production systems [34,39]. This suggests a distinction between how food business associates conceptualize safe food practices with inspectors, preferring a pragmatic and tangible approach over abstract ideals. Food business associates report discord with food safety regulations [34], and for some it gives rise to questioning the knowledge of inspectors, particularly in comparison to their own food safety knowledge [38]. This dichotomy of conceptualization may also explain why Ma et al. [41] found that food business associates largely categorize the severity of non-compliance descriptions differently to the design of a national inspection framework. The tension that arises from the conceptual dissonance, coupled with factors such as perception of inspection inadequacy, inspector incompetency and inconsistency may contribute to some food business associates questioning the need for government inspection at all [39].

### 3.3. Inspectors

#### 3.3.1. Inspection as a Behavioral Intervention

For inspectors, food safety inspection can be viewed as a means of intervention to motivate behavior change and promote safe food handling practices. These interventions can be directed toward food business associates while undertaking an inspection or can be targeted indirectly to influence market pressures and promote greater compliance via disclosure of results to consumers. In their research, Almanza et al. [42] found that a majority of inspectors felt disclosure of inspection results via the media would raise public awareness of food safety and would increase compliance of food businesses. Similarly, Newbold et al. [43] report inspector views that food safety inspection, coupled with public disclosure of results and more assertive enforcement practices may motivate improvement of food handling practices in food businesses. Other inspectors report more direct means of intervention such as applying food safety inspection as an opportunity to educate and inform food business operators of safe food handling practices [43]. These perspectives of inspection as an intervention suggest that the purpose of inspection is to address non-compliance of food businesses. The system being geared toward food businesses that are non-compliant and that are viewed to present a higher risk of causing foodborne illness [36].

#### 3.3.2. Inspection and Its Various Relationships with Risk

Risk is a concept that features regularly in the domain of food safety inspections. Yet the conceptualization of risk and the way it is applied is not universal amongst inspectors and regulatory authorities. For some inspectors, risk should form the basis for determining which food premises are inspected and how regularly this occurs [4,43]. To some, the frequency of inspection should be determined by risk calculated on food premises compliance history [43]. Here, risk conceptualization is focused on patterns of observed food handling behavior and compliance. For others, prioritizing premises for inspection with a basis on risk may consider the capacity of food producers to manage their food safety requirements inhouse, or may consider the number of persons potentially exposed to the food products [4]. This conceptualization of risk is focused on the reliability of internal assurance systems and reproducibility, to control the likelihood of foodborne illness. While a population-at-risk approach adopts a control of consequence, considering the extent of impact should a food contamination event occur. Further to scheduling and prioritization for inspections, risk can play a role in how an inspection is undertaken. Some inspectors recount past performance of food premises influencing their strategies for conducting an inspection, while others apply a risk based framework as a means of guiding their food safety inspection procedure and approach [36].

#### 3.3.3. Inspection as Collaboration

The approaches inspectors apply to food safety inspection suggests that inspection is more than an observational reporting process to them. Like food business associates, inspectors recognize inspection as a foundation for relationships with food business associates. Inspectors describe cooperation of food business associates as a desired objective when engaging in food safety inspection [44]. To foster this cooperation, inspectors will employ empathy and work to establish a personable relationship with food business associates [44]. These collaborative approaches to inspection offer a platform for inspectors to administer inspection as a means to immediate resolution of food safety issues. Some inspectors report a preference to fix issues during the inspection, rather than cite the non-compliance in their report [44]. For others, this approach is preferred because it allows them to take an educational approach and assist food business associates in problem solving [34]. However, the approach of collaborative problem solving is not always applied during inspections and can depend on the willingness of food business associates to adopt a learning perspective, and the extent to which inspectors trust that the food business associate will resolve the issue [34]. These approaches can also cause internal conflict for inspectors, particularly where inspectors strive to protect consumers with thoroughness and completeness in their identification of issues that could lead to foodborne illness [4].

#### 3.3.4. Inspection as a Multidimensional Evaluation

It is clear that to inspectors, food safety inspection involves more than determining compliance of food production premises and procedures with food safety regulations. Their evaluations may span dimensions such as relationships, trust, financial cost, and reasonableness. To some, it involves an evaluation of trust that food business associates will remedy non-compliances identified during the inspection [34]. For others, it can include an evaluation of reasonableness as to the costs that the food business is likely to incur in resolving non-compliances or meeting regulations [34]. Even the interpretation and application of food safety requirements may be subject to inspector evaluation of specific situations presented by food premises [34]. Accordingly Kaskela et al. [45] found that most inspectors in their study felt that inspection grading systems should have a small to moderate openness to interpretation. Yet as inspectors step beyond food safety regulations and apply professional discretion in reaching their decisions, they may expose themselves to challenges and disputes. For some inspectors, the reliance on regulatory systems and defined inspection procedures can provide them a defendable position, particularly when met with accusations of inconsistency, inequality, or inaccuracy [4].

#### 3.3.5. Inspection as Communication

To avoid dispute and discrimination, inspectors report a careful and deliberate approach to their communication and representation of inspection findings. Inspectors express that there are words they deem inappropriate for use in inspection reports [46]. These words may be inappropriate because they can have various interpretations, emotive connotations, may be unclear to consumers where reports are being disclosed, or may be misrepresentative [46]. Inspectors can also be challenged by standardized inspection reporting systems, particularly because these systems can oversimplify their findings, can lack detail and stifle expression, and again lead to misrepresentation [45]. Beyond the descriptive elements of inspection reports, inspectors also describe thoughtful ascription of inspection scores or grades. While for some this is to avoid enforcement obligations [11], others explain that they are mindful about what the grade could communicate to food business associates and consumers [4]. This corresponds to inspectors regularly avoiding extremes of grading systems, with intentions of communicating that premises are not without issues but also not an immediate health risk [4]. Inspectors also reflect on the impact grading systems can have as a communication method, identifying the limitations they impose on expression and representations of their findings for consumers when making dining decisions [47]. Although inspectors convey a desire to maintain expressiveness and representativeness in their communications about inspections, other influences such as resources and organizational structure may limit the extent that this can be achieved.

#### 3.3.6. Inspection as a Product of Resources, Structure and Regulatory System

For inspectors, resourcing can impose significant limitations on what their inspections achieve. Time is a constraint that leads some inspectors to adopt pragmatic approaches when writing inspection reports. For some, this translates to prioritizing non-compliances that are recorded in the inspection report and excluding those of lesser importance [46]. For others, time limitations can mean they do not achieve their desired level of expressiveness in writing their reports [46]. Constrained resources for inspectors may be a result of political interests favoring economic growth and business development over regulatory controls [4], but it may also result from poorly structured regulatory systems [48]. In his testimony before the United States Senate, Dyckman [48] identified that the large number of regulators involved in the United States’ food safety regulatory system was leading to costly inefficiencies, while also creating gaps in the way food safety inspections were conducted, their frequency, the regulations and enforcement applied, and duplication of effort between agencies. Some inspectors believe that food safety inspections should be more thorough and subject to regular auditing [49], yet the resources available to establish this standard of rigor may be insufficient in many cases. This could present notable consequences for inspectors as the nature of food safety regulations, statutes and constitutional rights in some jurisdictions can expose their inspection findings to legal contest by food businesses [4]. Furthermore, constrained resources may also have an impact on the extent of training inspectors are provided.

#### 3.3.7. Inspection as a Product of Inspector Training and Experience

Training and experience can have an impact on inspectors’ feelings of proficiency when inspecting and can shape their approaches to inspection. Training can predispose inspection and reporting styles that inspectors adopt [47]. For some, a lack of adequate training can lead to uncertainty and feelings of ineptitude in identifying foods that pose a risk to consumers [32]. Some inspectors feel that further training in detection methods and use of equipment to detect contaminated food is what they require to undertake their role more effectively [32], yet for others, competence and skill in inspection comes from other experiences in life. Experienced food safety inspectors recount how parenthood, teaching and real-world experience has strengthened their proficiency as an inspector, enriching their interpersonal skills and offering them more enlightened perspectives on the broader context of inspection [44]. Their reflection on their development as an inspector involves a transition from binary interpretations and rigid, system-driven decision making to a position of flexibility, understanding, autonomy, and discretion [34,44]. Experience in performing food safety inspection can also contribute to an inspector’s enlightenment and mastery in the undertaking, shaping the approach that inspectors choose to adopt while inspecting [36]. It is this transformational journey on a continuum of experience and mastery that appears to influence how inspectors view and attribute meaning to food safety inspection.

## 4. Discussion

Finding the meanings attributed to food safety inspection and applying a sociological lens is important as it offers the opportunity to explore what are society’s objectives and expectations of food safety inspection. It is important also because it allows for exploration of the social construction of problems, especially the criticisms often pitched at food safety inspection. By acknowledging the meanings society attribute to food safety inspection, it provides an opportunity to isolate values and constructs, allowing for inspection methods to be analyzed both in their presence and their absence. Where values and constructs are not isolated in this way, they present a chance that scientific rationality will be impeded by their influence.

While the findings of this review have identified a number of meanings attributed to food safety inspection, they also indicate that there are problems perceived with food safety inspections by society. These problems evolve around concepts such as compliance, and consistency in the application of food safety inspection. These problems are examined below through the isolation, inclusion, and exclusion of these values and constructs.

### 4.1. Consistency

#### 4.1.1. Consistency, Fairness, and Equality

A notable and common criticism of food safety inspection is that it lacks consistency. Claims of inconsistency appear to relate primarily to the analysis of inspection findings and the subsequent application of regulatory requirements [11,34,40,45,50,51,52]. Accordingly, this leads food business associates to describe the inconsistency of inspection outcomes as unfair and unreasonable, particularly with regard to their food business’ ability to compete in the marketplace [34]. Such inconsistencies may have financial and practical implications for food businesses, particularly where competitors are able to adopt simpler or cheaper methods in their food production while others are not [34]. However, beyond these practical implications, consistency is a concept that society appears to hold synonymous with values of fairness and equality [40,45]. Where inconsistency is perceived, moral obligations to uphold fairness and equality are also violated.

#### 4.1.2. Methodological Incongruence

In order to avoid inconsistency of results or measurements in positivist research, it is common practice to calibrate the instrument being used to take the measurement [53]. Food safety inspections are largely qualitative investigations, gathering data through observations, discourse, and document analysis [8]. Hence, rather than scientific or mechanical instrumentation, it is the inspector that is positioned as the instrument of research [53,54]. Consequently, the nature of qualitative inquiry, the means of establishing the quality of evidence, and the elements that are fundamental to establishing rigor in the research methods and findings differ considerably from those of quantitative research [55]. Concepts of consistency, replicability and reliability are not immediately transferable into the domain of qualitative research as it involves practices of interpretation rather than measurement [56]. Thus, the use of qualitative methods for undertaking food safety inspection preclude it from producing consistent results. Pursuit of consistent, replicable results using qualitative methods of inquiry is a methodological incongruence; the method of inquiry does not match the evidence sought [56,57]. Instead, qualitative research is best appraised in terms of trustworthiness, rather than in terms of reliability and replicability [58]. Trustworthiness relies particularly on the credibility of the research; confidence in accurate interpretation of data, largely as a product of methodological and procedural strength; consistency of the method to the research goal and transparency of the process of interpretation, and the believability rather than the consistency of the results [54,58,59].

#### 4.1.3. Situational and Experiential Nuances

One of the inherent values of adopting a qualitative approach to inquiry is that it identifies subtle situational and experiential nuances [60]. As such it can be expected that situational elements may influence the way inspectors apply inspection findings and regulatory requirements differently between food businesses. As Buckley [34] heard from inspectors, the variations of interpretation of food safety requirements between inspectors are highly circumstantial. Furthermore, as familiarity with food businesses for inspectors increases, food business associates report a greater sense of fairness of the inspection findings [35]. This is supported by findings of Kovács et al. [61] where repeat interaction between inspectors and food businesses result in inspection grade inflation. These findings suggest that situational elements such as familiarity, relationships, and trust, as well as compliance history of the food business, inspector perceptions and experience, and judgement in the absence of clear procedures may be considered in the food safety inspection and may lead to inconsistency of inspection results and in the application of regulatory requirements [9,11].

#### 4.1.4. Policy Implications of Consistency: Decoupling Concepts of Consistency

Although inconsistent inspection results may not fulfil society’s expectations for maintaining fairness and equality, satisfying these values are unlikely to be antecedent to preventing foodborne illness anyway. Upon examination in the absence of these societal values, no clear causal links present between the inspection outcome of one food business increasing the risk of foodborne illness at another food business, unless of course one is a supplier of food products to the other. Yet while consistency of inspection outcomes may not offer benefits in preventing foodborne illness, the consistency, and systematic application of methods for undertaking food safety inspection may [9,62]. It is important, however, that these two concepts of inconsistency be decoupled. Although they appear to be held synonymous by society, repeatability and systematism of methods are a key factor in food safety inspection resulting in prevention of foodborne illness [62], rather than the inspection findings and application of requirements. Hence, the rigorous application of qualitative research methods applied by food safety inspectors is an essential area for future research.

### 4.2. Compliance

#### 4.2.1. Virtue of Compliance

For consumers, there is a distinct relationship between violation of food safety requirements and violation of moral obligations by food businesses. For some consumers, tolerance for violations of food safety regulations is very low and is accompanied by feelings that violations should be met with strict enforcement action [24,29,31,33]. It appears that inspectors also carry a sense of how society values the concept of compliance, reporting that they manipulate inspection results to avoid enforcement actions and control their messaging [4]. Goss [63] and Makofske [13] also observed these manipulations, but as a benevolence amongst inspectors where inspection scores were often moved to a higher grading when close to a margin between grade categories. Accordingly, compliance is a concept that society seems to hold synonymous with virtuousness. Yet, the manner that compliance is conceptualized by society and the links of causality may be overly simplistic.

#### 4.2.2. Variables and Causality

Outbreaks of foodborne illness are influenced by more factors than compliance of a food business at the time of inspection. Human error has been identified as a key factor leading to incidents of foodborne illness [64]. While the compliance of a food business with food safety regulations has been found to relate to a reduced likelihood of food produced being microbially contaminated, and more generally to a reduced likelihood of causing outbreak of foodborne illness [62], consideration must be given to the likelihood that a food business may deviate from standard practices outside of inspection [65]. Hence, another common criticism of food safety inspection is that it only provides a snapshot in time [19]. Accordingly, compliance at the time of inspection must not be considered in isolation, but rather in conjunction with the ability of food businesses to maintain steady-state operations and minimize human error in ongoing operations.

#### 4.2.3. Policy Implications of Compliance: Truth before Virtue

In recognition of the wider influences on causation of foodborne illness, there are two concepts that must be revisited. The first is that the level of compliance as established at a food safety inspection should be considered as a general indicator, rather than impute certainty that foodborne illness will ensue. Such an approach recognizes that causation of foodborne illness extends beyond compliance and allows for food safety inspection to be applied as a risk-based model, where likelihood may now form part of the analysis. This transition from hazard focus to a risk focus demands that violation of food safety regulations can no longer be considered analogous for violation of moral obligations. The second concept is that the pursuit of virtuousness in the form of compliance must be reoriented to a pursuit of truthfulness. By reorienting these priorities, inspectors may be less inclined to manipulate inspection results, and intervention may be directed to food businesses that present the greatest risk of causing foodborne illness.

### 4.3. Strengths and Limitations

There are limitations to this review, particularly spanning from the lack of studies that directly capture beliefs and perceptions with regards to food safety inspection. Furthermore, no articles captured by the literature search exclusively investigate meanings attributed to food safety inspection directly, rather focusing on sub-elements such as views toward food safety inspection frameworks, disclosure of inspection results and preferences in communication style, methods applied to food safety regulation, or interpersonal interactions during food safety inspections. Due acknowledgement is made that while great effort has been expended to present these results in a balanced and faithful manner, as they have been drawn from research pursuing alternative foci of inquiry, some contextual depth and grounding may be foregone.

Despite these limitations, this review highlights the importance of identifying sociological factors when undertaking research into food safety inspection. It identifies a distinct limitation in the current body of knowledge surrounding food safety inspection, where values and logic are often left interlaced, and their dissension overlooked. The findings of this review highlight the importance of further research into meanings attributed to food safety inspection as a pre-requisite to more coherent research into food safety inspection methods.

## 5. Conclusions

Through critical examination of available literature, this review identified meanings attributed to food safety inspection by consumers, food business associates, and inspectors. Values, constructs, and meanings attributed to food safety inspection were then examined in light of common problems ascribed to food safety inspection: consistency and compliance. This examination demonstrated the influence of meanings, values, and constructs on how food safety inspection is realized, shaped, and rationalized. While limitations were encountered in the nature of the data available in the literature, this review highlights an important area for further research. Furthermore, it demonstrates that establishing the meanings attributed to food safety inspection is an imperative pre-requisite to any further meaningful research into food safety inspection and its effectiveness and method. Hence, by identifying and isolating values, constructs and meanings, food safety inspection can be examined with scientific rationality and in a more competent and percipient manner.
